# Examining the Validity of the World Health Organization's Long-Standing Hearing Impairment Grading System for Unaided Communication in Age-Related Hearing Loss

**DOI:** 10.1044/2018_AJA-HEAL18-18-0155

**Published:** 2019-10-16

**Authors:** Larry E. Humes

**Affiliations:** a Department of Speech and Hearing Sciences, Indiana University, Bloomington

## Abstract

**Objective:**

This review article overviews a presentation at the Hearing Across the Lifespan 2018 Conference, which examined the data from 5 data sets having pure-tone thresholds and functional measures of speech communication from relatively large groups of older adults to evaluate the validity of the long-standing World Health Organization (WHO) hearing impairment (HI) grading system.

**Design:**

This was a review of studies identified from the literature having both pure-tone audiometry and functional measures of speech communication from relatively large samples of older adults.

**Study sample:**

Three population or population–sample data sets and 2 clinical data sets were identified and included in the review.

**Results:**

As the WHO-HI grade progressed from “normal” to “severe” (insufficient data from older adults were available for the “profound” category), each step in this progression led to a significant difference in functional communication relative to all other WHO-HI grades. This was true for self-report measures of speech communication and direct measures of speech recognition in quiet and noise. Cohen's *d* effect sizes were moderate to very large between each successive step on the WHO-HI grading scale.

**Conclusions:**

The long-standing WHO-HI grading system, developed through expert opinion and adopted by WHO originally in 1991, is validated here with evidence from studies of functional communication in older adults. The WHO-HI grade system is compared to a proposed new WHO-HI grade system that introduces several changes to the grading system.

In 1991, the World Health Organization (WHO) convened an informal working group on the prevention of deafness and hearing impairment (HI; WHO, 1991). One of the key goals was to attempt to standardize the way in which severity of hearing loss was defined. This was critical to the gathering of evidence around the world regarding the prevalence of impaired hearing and deafness. Prior to developing plans to address a health care problem, one must be able to determine the pervasiveness of the problem, as well as population factors that might impact prevalence.

The long-standing WHO-HI grade system appears in Table 1, along with the presumed functional consequences for communication associated with each HI grade. With the definitions of impaired hearing from the WHO-HI grade system, epidemiologists could then go about the task of determining the prevalence and incidence of impaired hearing on a worldwide basis. In addition, the influence of key variables, such as age and gender, could also be determined (e.g., Stevens et al., 2013). Once prevalence was established, strategies to reduce or eliminate impaired hearing among the world's population could then be mapped out for future evaluation (e.g., Olusanya, Neumann, & Saunders, 2014).

**Table 1.** World Health Organization hearing impairment grades and presumed functional consequences.

Grade and corresponding audiometric ISO value^a^	Performance^b^
0 = No impairment, ≤ 25 dB	No or very slight hearing problems.
1 = Mild/slight, 26–40 dB	No problems in quiet with normal voice at 1 m. Hearing aids may be needed.
2 = Moderate 41–60 dB	Able to hear and repeat words using raised voice at 1 m. Hearing aids recommended.
3 = Severe, 61–80 dB	Able to hear some words when shouted into better ear. Hearing aids needed.
5 = Profound impairment, ≥ 81 dB	Unable to hear and understand even a shouted voice. Hearing aids may help, but additional rehabilitation needed.

a The audiometric dB HL (International Standards Organization) values are averages of values at 500, 1000, 2000, and 4000 Hz for the better ear.

b From WHO (1991).

Two considerations are noteworthy regarding the long-standing WHO-HI grade system. First, this WHO-HI grade system was established by expert opinion from the consensus of a panel of 14 international experts, rather than via available evidence. This is not surprising given that the WHO-HI grade system was established in 1991 when there was little or no large-scale evidence available to develop such a system. Second, the grading system is based on an average measure of hearing loss based on pure-tone thresholds. Since the publication of the original WHO-HI grading system in 1991, WHO (2001) redefined the very basis of bodily impairment, including HI, and its consequences. Within this framework, pure-tone audiometry may be a reasonable metric for the impairment of bodily structures or functions associated with hearing, but it may not be reflective of the associated impact on a person's *activity restrictions* or *the limits placed on their participation* in society, two key components of the WHO (2001) model. As shown in Table 1, progressively severe deficits in communication function are implied by the WHO-HI grade system. Evidence from functional measures of speech communication is needed, however, to establish the validity of the communication deficits associated with the severity of pure-tone hearing loss.

In this review, we attempt to fill this void in our knowledge regarding the validity of the long-standing WHO-HI grade system. Given the high prevalence of age-related hearing loss (ARHL) worldwide (WHO, 2012), we focused on the application of the WHO-HI grade system to this burgeoning group of adults with impaired hearing. (It should be noted that the WHO-HI grade system was designed to apply to all ages, from young children through older adults.) We obtained access to various data sets with a reasonable volume of data and which contained not only pure-tone thresholds but also at least one functional measure of communication performance, for older adults. Given the relative scarcity of such data, we accepted both self-report measures of hearing-related function and direct measures of speech recognition performance as functional measures of communication.

As noted, WHO has proposed a new WHO-HI grade system (Stevens et al., 2013). This proposed system lowers the boundary between “normal” and “mild” hearing loss to a better ear four-frequency pure-tone average (4fPTA; average of thresholds at 500, 1000, 2000, and 4000 Hz) of 20 dB HL from the original boundary at 25 dB HL. The proposed WHO-HI system also makes each subsequent step in the grade system uniform at 15 dB such that “moderate” hearing loss, the next step after mild, begins at 35 dB HL, rather than 40 dB HL as in the long-standing WHO-HI grade system. This is significant because WHO considers “disabling” HI to begin at the moderate level in both HI grade systems. Another change in the proposed WHO-HI grade system is the inclusion of a new grade or category, “moderately severe,” between the “moderate” and “severe” grades. Finally, the proposed new WHO-HI grade system also defined unilateral hearing loss operationally so that information could be gathered on this type of HI. Given the numerous changes from the long-standing original WHO-HI grade system established in 1991 and the proposed new WHO-HI grade system, we will also compare the two grade systems at the end of this review. Humes (2018) has presented a detailed evaluation of the validity of the proposed new WHO-HI grade system paralleling the evaluation of the long-standing original WHO-HI grade system presented at the Hearing Across the Lifespan (HEAL) 2018 conference and summarized here.

## Data Sets Examined and Methods

We have evaluated the application of the WHO-HI grade system to the data from three samples of the general population, two from the United States and one from Australia, and two clinical samples from the Department of Veterans Affairs (VA) Medical Centers in the United States. Two of the general population data sets were population studies, one of the residents of Beaver Dam, Wisconsin, who participated in the Epidemiology of Hearing Loss Study-2 (EHLS2; Cruickshanks et al., 1998; Nondahl et al., 1998), and one of the residents of the Blue Mountains region west of Sydney, Australia (Golding, Carter, Mitchell & Hood, 2004; Sindhusake et al., 2001). The third general population data set was from the 2011–12 National Health and Nutrition Examination Survey (NHANES; Center for Disease Control and Prevention, 2011). NHANES is a survey that combines interviews and physical examinations for a nationally representative sample of about 5,000 Americans each year. For this overview of the HEAL 2018 presentation, the NHANES data are not considered due to their less thorough assessment of hearing and communication difficulties. These data are included, however, in the more detailed review of the proposed new WHO-HI grade system (Humes, 2018).

The two VA data sets represented reasonably large samples from clinical populations from the United States. One study (Wilson, 2011) included participants primarily from Upper East Tennessee, whereas the other (Williams-Sanchez et al., 2014) included participants from Florida, Tennessee, and California. The Hearing Handicap Inventory for the Elderly–Screening version (HHIE-S) data obtained by Wilson (2011) were not published in that article, but the author was granted access to these data by the investigator (R. Wilson).

For four of the five data sets, all except the EHLS2, we were able to obtain access to the de-identified raw data for analyses. For the EHLS2, a collaborator from that study provided the analyses shown below.

For each data set, we calculated the 4fPTA by obtaining the means of the thresholds at 500, 1000, 2000, and 4000 Hz for each ear, and then selected the better ear 4fPTA for each participant. Ear-specific and better ear 4fPTAs were used to then assign a WHO-HI grade as follows: (a) normal, ≤ 25.50; (b) slight/mild, 25.51 to 40.5; (c) moderate, 40.51 to 60.5; (d) severe, 60.51 to 80.5; and (e) profound, ≥ 80.51 dB HL. Those meeting the proposed new WHO definition of unilateral hearing loss, 4fPTA < 20 dB HL in the better ear and ≥ 35 dB HL in the worse ear, were removed prior to analysis as unilateral HI is not a typical characteristic of age-related hearing loss. Across all five studies, less than 1.5% of each study sample met this definition of unilateral hearing loss and were excluded from further analyses.

The resulting WHO-HI grade then became the primary independent variable used in ensuing analyses of variance with the dependent variables being the functional measures of hearing available. Prior to examining the effects on functional measures of hearing, age differences between WHO-HI grades were evaluated. Because we were interested in the effects of the grade of HI on communication performance for older adults, age was a covariate in all analyses of variance. “Older adults” were generally defined as age > 50 years, but 70%–80% of the data from most of the data sets examined here were from participants in their 60s and 70s. For the NHANES data set, only individuals ranging from 50 to 69 years of age were included as 69 years old was the maximum age included in that national survey. The EHLS2 data set, on the other hand, had a broader age range included than the other data sets (53–95 years) and had a higher percentage of individuals in their 80s than any of the other data sets. Significant effects of WHO-HI grade were followed up by posthoc *t* tests at *p* < .05 for pairwise comparisons where appropriate. Given the relatively large samples, even arithmetically small differences between WHO-HI grades may be statistically significant. To get a better idea of the practical significance of differences between WHO-HI grades, we also calculated Cohen's *d* to get an estimate of the effect size as one progressed through each successive step on the WHO-HI grade system.

## Results

### Self-Report Measures

Of the various self-report measures reviewed in the presentation at the HEAL 2018 conference, the HHIE-S (Ventry & Weinstein, 1982, 1983) was the standardized measure most commonly employed. The HHIE-S is composed of 10 items describing social or emotional reactions to hearing loss, and the individual indicates whether he or she has had this reaction. Responses are scored as follows: “no” = 0 points, “sometimes” = 2 points, and “yes” = 4 points. The HHIE-S scores are total scores summed across all 10 items and range from 0 to 40 with higher scores indicating greater hearing handicap.

**Figure 1.**
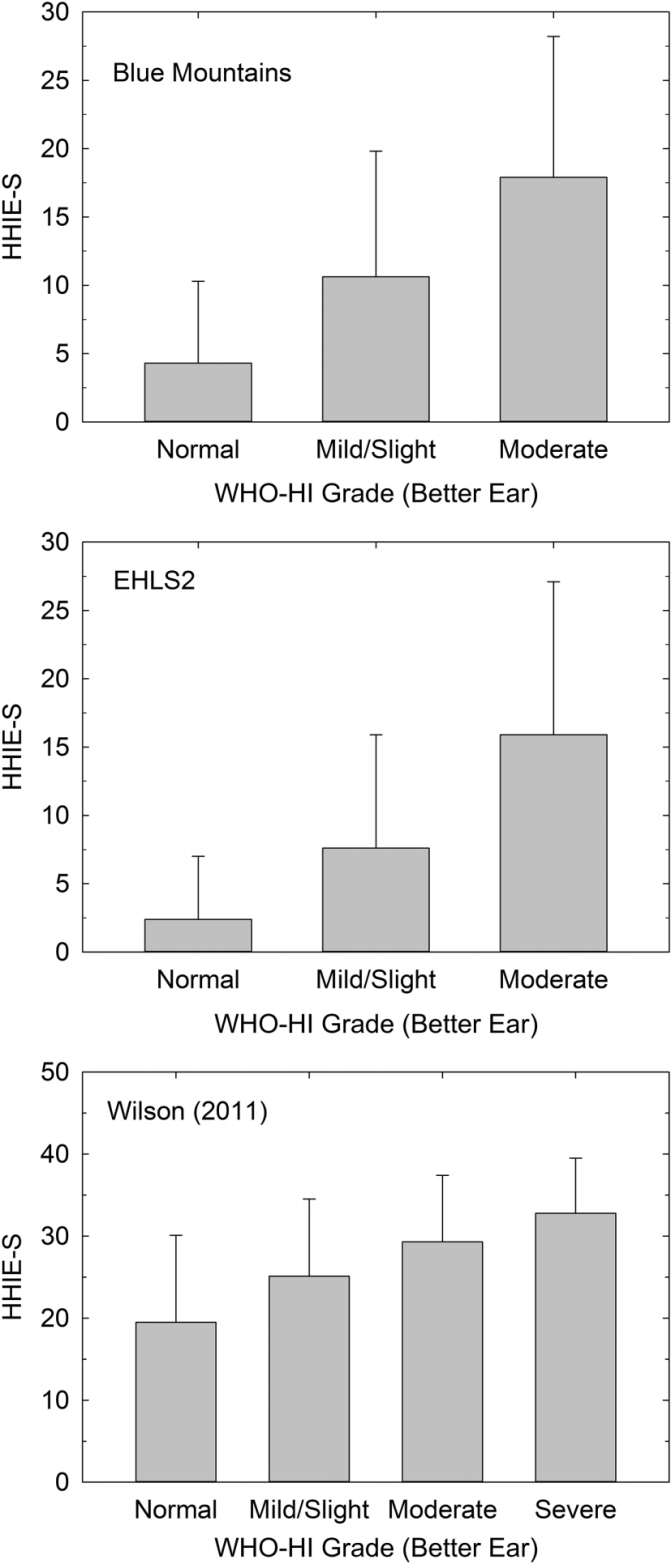
Means (+1 *SD*) for the Hearing Handicap Inventory for the Elderly–Screening version (HHIE-S) as a function of World Health Organization (WHO) hearing impairment (HI) grade for the better ear from the Blue Mountains Hearing Study (top), Epidemiology of Hearing Loss Study-2 (EHLS2; middle), and Wilson (2011; bottom).

HHIE-S scores were available from 2,756 Blue Mountains Hearing Study (BMHS) participants, 2,630 EHLS2 participants, and 2,754 individuals from the VA clinical study by Wilson (2011). Figure 1 provides the means (+1 *SD*) for the HHIE-S from each of these three studies plotted as a function of the better ear WHO-HI grade. As expected, the two population studies of older adults did not have many cases beyond a WHO-HI grade of moderate, but the VA clinical sample included many more participants with severe WHO-HI grades, enough (*N* = 70) to warrant inclusion of the data for that WHO-HI grade in the bottom panel of Figure 1. Note also that the mean HHIE-S scores for a given WHO-HI grade are considerably higher in the VA clinical sample (bottom panel; ordinate maximum of 50), indicating greater perceived hearing handicap, than either of the population studies (top two panels; ordinate maximum of 30). This is expected given the decidedly different nature of the clinical and population samples. Whereas population studies attempt to assess everyone in a given population, the VA clinical data are from participants who reported to the clinic because they were experiencing hearing difficulties. More pertinent to the focus of this review, in all three samples, there was a significant effect of WHO-HI grade on HHIE-S score, smallest *F*(4, 2749) = 138.0, *p* < .001, age as covariate, and all Bonferroni-corrected pairwise comparisons between WHO-HI grades in a given study were significantly different (*p* < .05).

### Speech Recognition Measures

**Figure 2.**
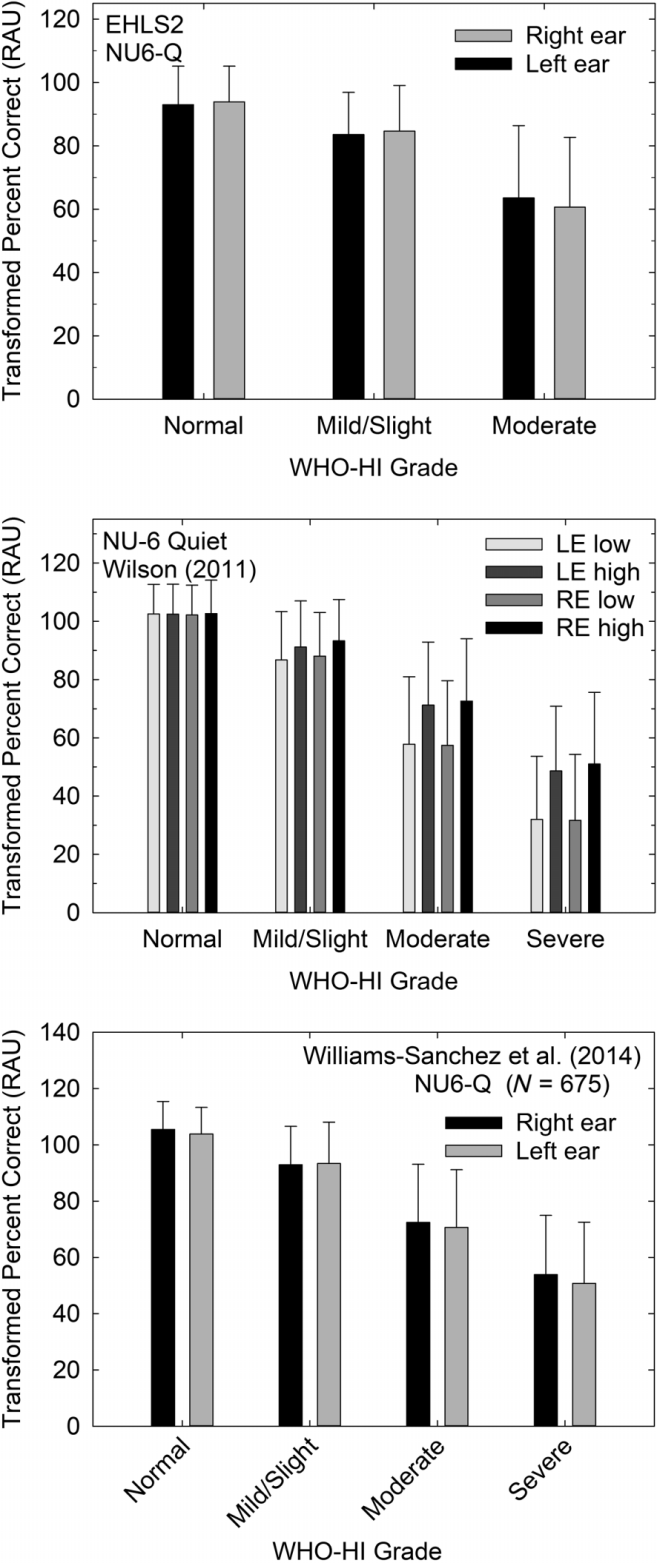
Means (+1 *SD*) for the rationalized arcsine unit (RAU)-transformed percent correct scores for the Northwestern University Auditory Test No. 6 (NU-6) speech recognition test from the Epidemiology of Hearing Loss Study-2 (EHLS2; top), Wilson (2011; middle), and Williams-Sanchez et al. (2014; bottom) data sets. Data for each ear plotted separately as a function of the ear-specific World Health Organization (WHO) hearing impairment (HI) grade in each panel. Data from Wilson in the middle panel are for all four combinations of ear (right ear [RE], left ear [LE]) and presentation level (low, high) used in that study.

Several ear-specific measures of speech recognition were also obtained under headphones from several of the population and clinical studies examined. The data from all of these measures and studies are reviewed in detail in Humes (2018) relative to the proposed new WHO-HI grade system. Here, this review of the long-standing WHO-HI grade system focuses on the two speech recognition measures obtained most frequently and using the same procedures and materials across studies. The speech stimuli were the Northwestern University Auditory Test No. 6 (NU-6) monosyllables spoken by a female talker (Wilson, Zizz, Shanks, & Causey, 1990). For the scores obtained in quiet, the same recorded speech materials were used for the EHLS2, Wilson (2011), and Williams-Sanchez et al. (2014) data sets. The percent correct scores were transformed into rationalized arcsine units (Studebaker, 1985) prior to analyses, and the means (+1 *SD*) from each study appear in Figure 2. Data for each ear, and for the Wilson (2011) data, for each presentation level, were analyzed separately via univariate analyses of variance, each adjusted for age. Because the data were obtained under earphones for the right and left ears separately, the ear-specific WHO-HI grade was used to analyze the data, rather than the better ear WHO-HI grade that was used in the prior analyses of the HHIE-S self-report measure.

For the EHLS2 data (top panel), subsets of participants completed measures of open-set speech recognition for monosyllables presented under headphones in quiet (*N* = 928 for left ear and *N* = 1,674 for right ear). The speech presentation levels were a moderate sensation level (36 dB) relative to the pure-tone threshold at 2000 Hz to minimize the effects of high-frequency inaudibility on performance (Wiley et al., 1998). The effects of WHO-HI grade on performance in quiet were significant for both right and left ears, smallest *F*(4, 923) = 124.9, *p* < .001, with performance decreasing as the severity of the HI increased. All pairwise comparisons of the word recognition scores differed significantly (*p* < .05) between WHO-HI groups.

For the Wilson (2011) VA data set, shown in the middle panel of Figure 2 for 2,991–3,131 participants, in addition to having ear-specific word recognition scores, scores were available for two presentation levels: (a) low presentation level of 80 dB SPL for those with a three-frequency (500, 1000, and 2000 Hz) pure-tone average ≤ 40 dB HL or 90 dB SPL for all other participants and (b) a high presentation level, 24 dB above the low level (either 104 or 114 dB SPL). Four separate univariate analyses of variance were performed, each corrected for age, to examine the effect of WHO-HI grade on word recognition performance in quiet. For all four measures of word recognition in quiet (left/right × low/high), significant effects of WHO-HI grade were observed, smallest *F*(3, 3005) = 398.7, *p* < .001, age adjusted. All post hoc pairwise, Bonferroni-corrected comparisons among WHO-HI groups were also significant (*p* < .05). In general, as hearing loss severity progressed from normal to severe, word recognition performance in quiet declined in both ears and for both presentation levels.

The bottom panel of Figure 2 shows the data for NU-6 monosyllabic words presented in quiet at a moderate sensation level designed to maximize word recognition performance for unaided listening from Williams-Sanchez et al. (2014; *N* = 670−675). Univariate analyses of variance, controlling for age, revealed a significant effect of WHO-HI grade on word recognition in quiet for the right ear, *F*(3, 670) = 121.7, *p* < .001, and the left ear, *F*(3, 665) = 124.0, *p* < .001. Post hoc pairwise, Bonferroni-corrected comparisons among WHO-HI grades revealed that each group differed significantly (*p* < .05) from all other groups for both ears.

**Figure 3.**
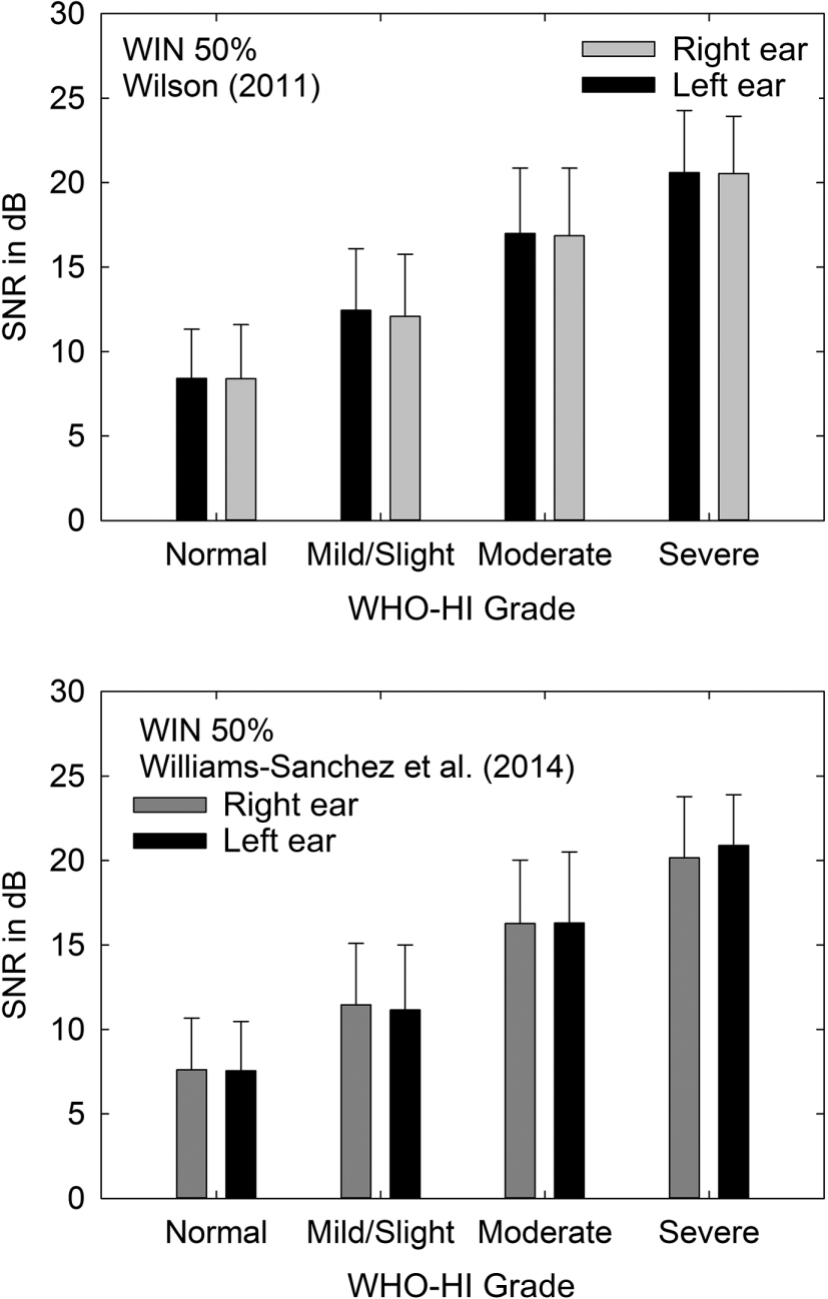
Mean (+1 *SD*) speech-to-noise ratios (SNR) in dB plotted as a function of World Health Organization (WHO) hearing impairment (HI) grade for clinical data sets from Wilson (2011, top) and Williams-Sanchez et al. (2014, bottom). Data are shown separately for the right and left ears, and ear-specific WHO-HI grades are indicated on the *x*-axis. WIN = words-in-noise.

In addition to speech recognition testing in quiet, several of the studies reviewed here also tested speech perception in noise. Test materials and conditions were more heterogeneous across studies for speech recognition in noise, but two of those studies made use of the same adaptive words-in-noise (WIN; Wilson, 2003) test. The words used in the WIN are a subset of the same NU-6 monosyllabic words used in quiet; those word stimuli found to yield sufficiently homogeneous performance to be used in an adaptive test procedure. In this adaptive procedure, a competing background of multitalker babble is mixed with the monosyllabic word stimuli in the same ear and the speech level needed to achieve 50% correct performance in that background is established. This yields the speech-to-noise ratio (SNR) corresponding to 50% correct. The mean (+1 *SD*) SNRs from Wilson (2011; *N* = 3,252–3,259) and Williams-Sanchez et al. (2014; *N* = 511–521) are plotted in Figure 3 as a function of WHO-HI grade with all measures again being ear-specific given earphone presentation of the speech stimuli. Univariate analyses of variance, one for each study and each ear, all corrected for age, were performed, and all four revealed significant effects of WHO-HI grade, smallest *F*(3, 506) = 105.7, *p* < .001. For both data sets, post hoc pairwise, Bonferroni-corrected comparisons among groups showed that each group differed significantly (*p* < .05) from all other groups. In general, as WHO-HI grade increased from normal to severe, the SNR needed to achieve 50% correct word recognition also increased. As expected, given similar participant samples and study methods, the actual SNRs obtained in both of these clinical VA data sets are very similar.

### Cohen's d

Given the relatively large sample sizes in the various data sets reviewed above, it is possible for small group differences to emerge as statistically significant differences. Recognizing this limitation, Cohen (1988) proposed a metric, *d*, to better interpret “effect sizes.” Using this metric, which has since been identified as Cohen's *d*, *d* values of 0.2, 0.5, and 0.8 have been labeled as *small, medium, *and *large* to aid in interpretation of group differences. We extended the boundaries by ± 0.15 for each of these effect-size categories and then tallied the number of small, medium, and large effect sizes observed. For the set of 70 Cohen's *d* values calculated across all five studies reviewed, 3% were small, 30% moderate, and 67% large. Thus, the changes in speech communication function between adjacent steps on the WHO-HI grade scale are robust effects and further support the validity of the HI categories formed.

## Discussion

This overview of the review presented at the HEAL 2018 examined the validity of the WHO-HI grade system across three relatively large population or population sample studies and two comparably large clinical studies that included measures of pure-tone hearing thresholds and some type of functional assessment of communication. The primary conclusion from this review is that there is good validity for the original WHO-HI grade system as established by evidence from relatively large population and clinical studies. Specifically, there are significant changes in functional communication among older adults as the WHO-HI grade progresses from slight/mild through severe. Most often, moreover, the significant changes in a data set were evident for each of several functional measures available from that sample. For example, this pattern was evident here in the self-report HHIE-S data, the speech recognition in quiet scores, and the WIN SNRs for the data from Wilson (2011). (The same held true for the data from the BMHS and EHLS2 population studies, although all the corresponding data from those studies are not presented in this overview.) Across all studies reviewed, there were insufficient data for those with “profound” hearing loss (4fPTA > 80 dB HL) to evaluate the validity of that WHO-HI grade. However, there are few older adults with profound hearing loss in their better ear.

The progression from slight/mild through severe WHO-HI grades for older adults was not just statistically significant from one grade to the next, but also typically manifested medium to large Cohen's *d* effect sizes in most cases. Only 3% of the *d* values computed here would be considered by Cohen (1988) to be small (0.2 ± 0.15).

This review provides evidence from relatively large population and clinical samples that validates the long-standing WHO-HI grade system for its application to older adults with ARHL. Extensions to other populations differing in age or in nature of the HI from those examined here require separate evaluations. Nonetheless, the WHO-HI grade system appears to offer a valid grading of the severity of communication difficulties in the large and growing population of older adults, including those with ARHL.

**Figure 4.**
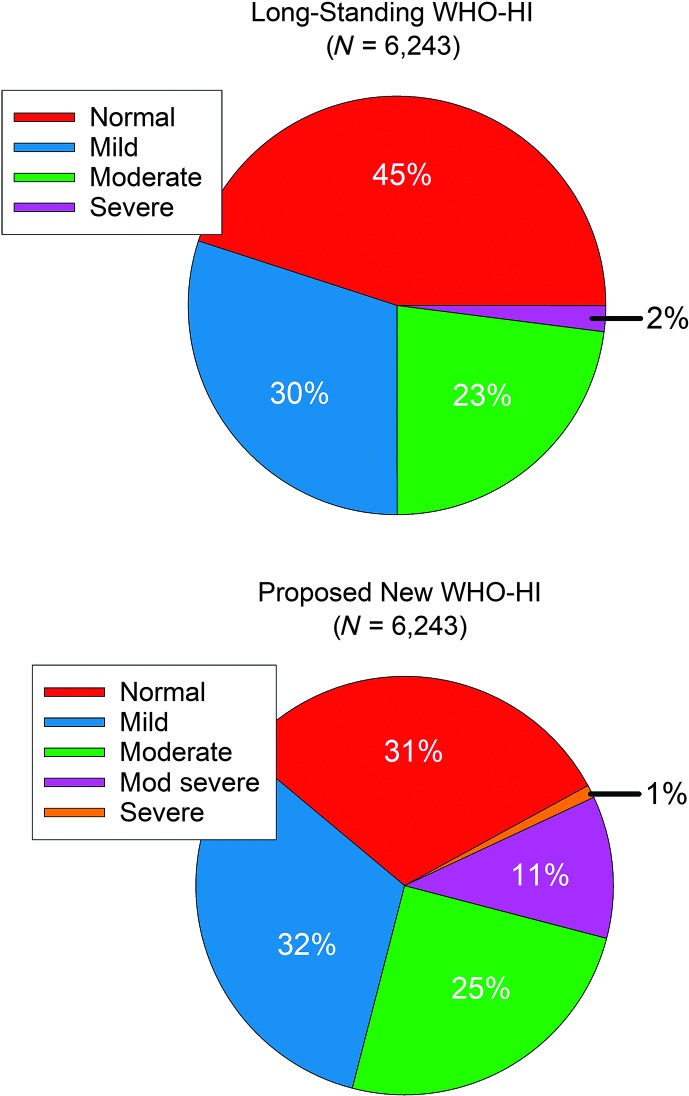
Pie charts showing the percentages of 6,243 individuals from the combined Blue Mountains Hearing Study and Wilson (2011) data sets who were classified into various World Health Organization (WHO) hearing impairment (HI) grades. The top pie chart provides the results for the long-standing WHO-HI grade scale, whereas the bottom pie chart provides the corresponding results for the proposed new WHO-HI grade scale.

As noted, Humes (2018) provides a detailed evaluation of the validity of the proposed new WHO-HI grade using the same data sets and approach described for this overview of the validity of the long-standing WHO-HI grade system. Also noted previously, the proposed new WHO-HI grade system basically shifts the boundaries lower for the first three WHO-HI grades, normal, mild, and moderate, to make room for an additional intermediate WHO-HI grade, moderately severe. We pooled the HHIE-S scores from one population study, BMHS (Golding et al., 2004), and one clinical study, Wilson (2011), to form a combined data set of 6,243 individuals (following elimination of the small number of participants with unilateral hearing loss). The pie charts in Figure 4 illustrate the resulting partitioning of the same 6,243 individuals into the corresponding WHO-HI grades. The most striking difference between the two pie charts is the reduction, as expected, in the percentage of individuals categorized as “normal” by the proposed new grade system. Whereas the long-standing WHO-HI grade system classified 45% of the combined sample as normal, this was reduced to 31% for the proposed new WHO-HI grade system. By comparing the other WHO-HI grades in the two pie charts, this reduction in those categorized as normal by about 14 percentage points accommodates the addition of 11% constituting the newly proposed moderately severe WHO-HI grade. This is not to say that the 11% considered to have moderately severe HI in the lower pie chart were previously within the normal group in the top pie chart. Rather, there is a cascade of shifts in HI categories of about 11%–15% from normal to mild (*N* = 883), mild–moderate (*N* = 741), and moderate to moderately severe (*N* = 601) when comparing the original to the proposed WHO-HI classifications.

Another notable consequence of the proposed shift from the long-standing to the new WHO-HI grade system is the increase in those considered to have disabling HI, defined by WHO as those with at least moderate impairment in the better ear. Whereas 25% in the top pie chart met the original WHO definition for disabling HI, 37% would do so under the proposed new WHO-HI grade system.

**Figure 5.**
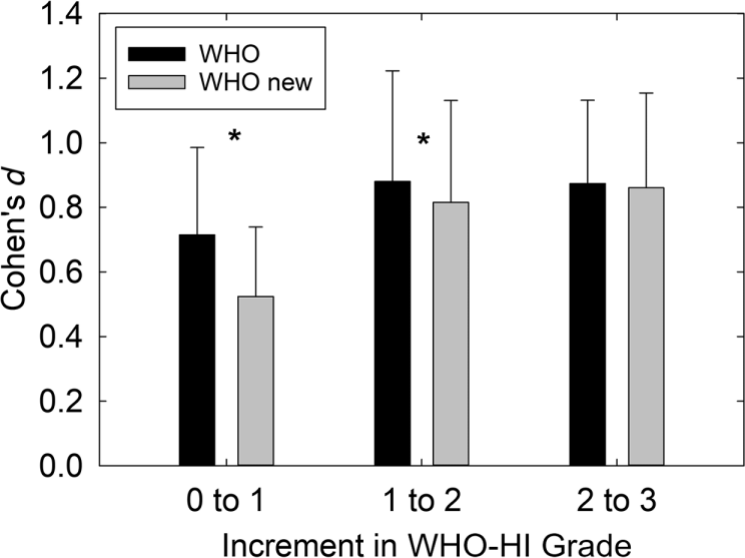
Mean (+1 *SD*) Cohen's *d* values for the original World Health Organization (WHO) hearing impairment (HI) grade system (WHO) and the proposed new WHO-HI grade system (WHO new) plotted as a function of the change in grade: from 0 to 1 (normal to mild), 1 to 2 (mild to moderate), and 2 to 3 (moderate to severe for the original WHO-HI and moderate to moderately severe for the proposed new WHO-HI system).

It is difficult to compare the two WHO-HI grade systems to establish which might be preferred. Lowering the boundary between normal and mild HI in the proposed new WHO-HI grade system is an attempt to recognize the significant consequences of even slight hearing loss on communication and function. Interestingly, the notion of lowering the boundary for normal to 20 from 25 dB HL was noted for consideration in the description of the original WHO (1991). The boundary between normal and mild HI has been a point of debate for some time. Of course, the risk in lowering that boundary lies in overidentifying those with impaired hearing in the process. One way to assess the robustness of boundaries may be through examination of the effect sizes for functional measures of communication across successive boundaries on each WHO-HI grade scale. As noted above, only 2 of 70 (3%) such Cohen's *d* values would be considered small (0.05 < *d* < 0.35) for the long-standing WHO-HI grade system. By comparison, 11 of 94 (12%) of the Cohen's *d* values were small for the proposed new WHO-HI grade system. Interestingly, 8 of the 11 small Cohen's *d* values observed for the proposed WHO-HI grade system occurred at the step from normal (0) to mild (1) in the system. Figure 5 compares the mean (+1 *SD*) Cohen's *d* values as each WHO-HI grade system progresses from normal to mild (0 to 1), mild–moderate (1 to 2), and moderate to severe or moderate to moderately severe (2 to 3). Paired-samples *t* tests revealed that the differences in *d* values at the 0 to 1 and 1 to 2 steps in the WHO-HI grade system differed significantly (*p* < .05) with the proposed new grade system having lower *d* values. Nonetheless, it is important to note that both grade systems show mean *d* values for each scale step that would be considered moderate to large effect sizes (*d* > 0.5).

Lowering the boundary between normal hearing and mild HI, however, is not the only change in the proposed new WHO-HI grade system. As noted, the increments between steps on the grade scale were made uniform at 15 dB and, as a result, another intermediate category, moderately severe, was needed. The two grade systems realign at the profound impairment grade as this is defined as better ear 4fPTA greater than 80 dB HL in both systems. Table 2 presents the categorization of 6,243 cases in the combined BMHS population and Wilson (2011) VA clinical data sets by the long-standing WHO-HI grade system (columns) and the proposed new WHO-HI grade system (rows). Another way in which to compare the two WHO-HI grade systems would be to see if the various subgroups in Table 2 differ from one another on a functional measure of communication, such as the HHIE-S, a measure used in both the BMHS and Wilson studies. For example, note in Table 2 that 883 of the 2,793 cases designated as normal on the original WHO-HI grade scale are classified as mild in the proposed new WHO-HI grade. Do these two subgroups, both classified as normal in the original WHO-HI grade system, differ significantly on a functional measure of communication like the HHIE-S? Similarly, do the 883 with differing HI grades in the two systems differ functionally from the 1,136 who were classified as mild by both the current and proposed WHO-HI grade systems?

**Table 2.** Tallies of World Health Organization (WHO) hearing impairment (HI) grade classifications, original system (columns) versus proposed new system (rows).

Proposed WHO-HI grade	Original WHO-HI grade			
	Normal	Mild	Moderate	Severe
Normal	1,910	0	0	0
Mild	883	1,136	0	0
Moderate	0	741	836	0
Moderately severe	0	0	601	78
Severe	0	0	0	58

*Note.* Data were from the Blue Mountains and Wilson (2011) data sets combined with unilateral cases and those with profound impairment (*N* = 14) deleted. Total *N* = 6,243.

**Figure 6.**
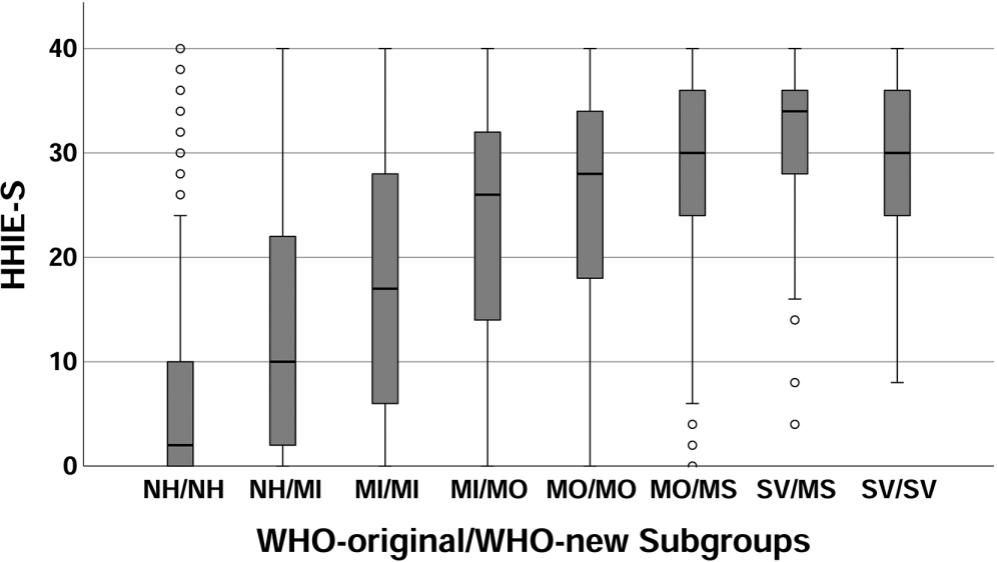
Boxplot showing medians (black horizontal line in each grey box), interquartile range (grey box), range (error bars), and outliers (circles) for the Handicap Inventory for the Elderly–Screening version (HHIE-S) for each of the eight World Health Organization (WHO) hearing impairment (HI) classification subgroups with nonzero entries in Table 2. NH = normal hearing; MI = mild; MO = moderate; MS = moderately severe; SV = severe.

To address these questions, participant subgroups were formed based on the eight WHO-HI grade combinations with nonzero entries in Table 2. Figure 6 presents a boxplot for the HHIE-S scores for the eight WHO-HI-grade subgroups. Visual inspection of these data in Figure 6 shows a steady increase in HHIE-S score from the subgroup considered to be normal (NH) on both WHO-HI grade scales (subgroup NH/NH, far left) through the subgroup considered to have moderate (MO) HI on both scales (subgroup MO/MO). At more severe impairments, those subgroups to the right of MO/MO in Figure 6, the median HHIE-S score appears to level out. These visual trends were confirmed through univariate analysis of variance for the HHIE-S scores as a function of WHO-HI subgroup with age as a covariate. There was a significant effect of WHO-HI subgroup on HHIE-S score, *F*(7, 5537) = 709.4, *p* < .001. Post hoc Bonferroni-corrected *t* tests for all paired comparisons revealed that the three rightmost subgroups did not differ significantly from one another (*p* > .76), but all other paired comparisons yielded significant differences (*p* < .05). Notice that two of the three nonsignificant differences in functional communication, at least as measured by the HHIE-S, involve the newly proposed moderately severe HI category. Neither group labeled as having moderately severe WHO-HI grades in Figure 6 (MO/MS and SV/MS subgroups) differed significantly from those categorized as severe in both WHO-HI grade systems (SV/SV subgroup). This evaluation, based on the HHIE-S, questions the validity of the moderately severe category as a distinct grade of HI separate from that of severe. Unfortunately, the HHIE-S was the only measure in common to the BMHS and Wilson (2011) studies. Further evaluation of the two WHO-HI grade systems must await the availability of additional data on the communication function of those classified with various degrees of HI.

Finally, both the original and the proposed new WHO-HI grade systems base their classification of impaired hearing on pure-tone audiometry, and there may be reason to question this metric of sensory impairment. Although the widespread availability of low-cost pure-tone audiometry and the ease of obtaining ear-specific measures, as well as comparison to a large volume of historical data, argue in favor of the continued use of pure-tone audiometry to define impaired sensory function, there may be reasons to reconsider this choice in future studies. For example, there is active investigation of the prevalence of auditory synaptopathy (Kujawa & Liberman, 2009) and possible concomitant “hidden hearing loss” in humans (e.g., Guest, Munro, Prendergast, Millman, & Plack, 2018; Hickox, Larsen, Heinz, Shinobu, & Whitton, 2017), which challenges the premise that normal hearing implies normal peripheral sensory processing. Furthermore, as noted above, the agreement between pure-tone thresholds and self-report measures of communication or hearing handicap is such that these two sets of measures overlap by about 50%; that is, knowing either metric can explain about 50% of the individual differences in the other. Thus, knowing a person's pure-tone audiogram only partially explains the functional consequences of hearing loss on that person's daily function as captured by self-report measures, such as the HHIE-S. One could argue, therefore, that the self-report measure should be used instead of pure-tone audiometry to capture impaired communication. “Impaired communication,” on the other hand, is much broader than impaired hearing, the impairment of a bodily function. In the WHO-ICF model (WHO, 2001), pure-tone audiometry offers a viable metric for impaired bodily function, in this case impaired hearing, and this model also acknowledges that impaired bodily function is only one contributor to the ensuing activity limitations and participation restrictions. Many environmental factors (social context, physical environment, etc.) and personal factors (age, cognitive function, personality, etc.) also contribute to the activity limitations and participation restrictions experienced by a person with impaired hearing. To the extent that the self-report measure captures the domains of activity limitations and participation restrictions, or “impaired communication and its consequences,” then the agreement between measures of bodily function, such as pure-tone audiometry, and broader self-report measures are expected to be less than perfect.
